# *Chaetomorpha linum* Extract as a Source of Antimicrobial Compounds: A Circular Bioeconomy Approach

**DOI:** 10.3390/md22110511

**Published:** 2024-11-13

**Authors:** Roberta Barletta, Alfonso Trezza, Michela Geminiani, Luisa Frusciante, Tommaso Olmastroni, Filomena Sannio, Jean-Denis Docquier, Annalisa Santucci

**Affiliations:** 1 Department of Biotechnology, Chemistry & Pharmacy, University of Siena, Via A. Moro, 53100 Siena, Italy; r.barletta@student.unisi.it (R.B.); geminiani2@unisi.it (M.G.); luisa.frusciante@unisi.it (L.F.); tommaso.olmastroni@student.unisi.it (T.O.); annalisa.santucci@unisi.it (A.S.); 2 SienabioACTIVE, University of Siena, Via Aldo Moro, 53100 Siena, Italy; 3 Department of Medical Biotechnologies, University of Siena, Viale Bracci 16, 53100 Siena, Italy; filomena.sannio@unisi.it (F.S.); jddocquier@unisi.it (J.-D.D.); 4 ARTES 4.0, Viale Rinaldo Piaggio, 34, 56025 Pontedera, Italy

**Keywords:** *Chaetomorpha linum*, circular bioeconomy, antimicrobial activity, *Enterococcus faecalis*, molecular modeling, docking simulations, molecular dynamics, MIC

## Abstract

The circular bioeconomy is currently a promising model for repurposing natural sources; these sources include plants due to their abundance of bioactive compounds. This study evaluated the antimicrobial properties of a *Chaetomorpha linum* extract. *Chaetomorpha linum* is an invasive macroalga from the Orbetello Lagoon (Tuscany, Italy), which grows in nutrient-rich environments and has been forming extended mats since 2005. The biomass is mechanically harvested and treated as waste, consuming considerable manpower and financial resources. As a potential way to increase the value of such waste, this study found that *C. linum* extract (CLE) is a source of antimicrobial compounds. The phytochemical characterization of the extract revealed the predominant presence of palmitic acid, a fatty acid with known antimicrobial activity. Based on such findings, four bacterial species of high clinical relevance (*Enterococcus faecalis*, *Staphylococcus aureus*, *Pseudomonas aeruginosa*, and *Escherichia coli*) were tested, revealing a notable antibacterial activity of the extract on *Enterococcus faecalis* (MIC, 32 μg/mL). Computational analyses identified a potential *Enterococcus faecalis* molecular target for palmitic acid, offering molecular insights on the interaction. This study presents a comprehensive in vitro and in silico approach for drug and target discovery studies by repurposing *C. linum* as a source of antimicrobial bioactive compounds.

## 1. Introduction

Seaweeds, the common name for marine macroalgae, and their extracts have become relevant for the development of nutraceutical products due to their richness in bioactive molecules. Indeed, a previous research-proven multitude of biological activities from seaweed makes them appealing to the pharmaceutic industry as a precious source of raw materials [1,2,3,4].

Moreover, interest in plant-based bioactive compounds is rising due to their abundance, large availability, and economic relevance [5,6].

Despite their long engagement in the food and feed industries [7], seaweed’s potential as a source for nutraceutical and medicinal applications remains largely unexplored, although they possess strong properties. Indeed, the literature body demonstrated that macroalgae offer protection against chronic diseases, such as allergic diseases, due to their high content of lipids, proteins, polysaccharides, fatty acids, and antioxidants [4,8,9,10]. Additionally, biomass removal from invaded areas stands as a valid supply source.

*Chaetomorpha linum* (Müller) Kützing (Basy-onim: *Conferva linum* O.F. Müll.) is a macroalgae species from the *Chlorophyceae* family, thriving in eutrophic conditions and forming dense mats that are difficult to remove, as seen in the Orbetello Lagoon (Tuscany, Italy) [11]. These mats, covering roughly 400 hectares, withstand mild winters, affecting local ecosystems and industries, such as fish farming. Therefore, they are treated as waste, and the biomass is mechanically harvested, with considerable manpower and financial burden. Between 2002 and 2006, an average of over 27,000 tons of macroalgae was annually removed from the lagoon at a high cost for municipalities [11,12].

A significant challenge is represented by the necessity of innovative applications for this macroalga as, contrastingly to other macroalgae from Orbetello Lagoon, it is not exploitable for biodiesel or bio-oil production [13,14]. From this perspective, this study was conducted, alongside the principles of a circular bioeconomy.

Unlike a linear bioeconomy, where natural resources are heavily consumed, a circular economy promotes environmental sustainability by focusing on resource reuse and recycling, which helps to reduce human impact and pollution. In scientific research, the circular bioeconomy is key in turning biological waste into sustainable products [15,16,17,18].

In this context, a joined in vitro and in silico pipeline was developed to investigate the antimicrobial activity of the hydroalcoholic *Chaetomorpha linum* extract.

Such an extract was previously characterized in one of our works. It revealed many bioactive compounds, including lipids, amino acids, terpenoids, and flavonoids, with fatty acids (palmitic acid, oleic acid, and stearidonic acid) predominant [18]. Fatty acids are known not only for their critical biological roles—such as maintaining membrane fluidity, providing energy storage, and serving as precursors for bioactive lipids involved in inflammation and immunity—but also for their antimicrobial activity.

Among the unsaturated fatty acids, two classes can be distinguished: monounsaturated fatty acids (MUFAs) and polyunsaturated fatty acids (PUFAs). Monounsaturated fatty acids (MUFAs) contain a single double bond in their carbon chain, which gives them unique stability and health benefits, including supporting cardiovascular health by improving lipid profiles [19]. In contrast, polyunsaturated fatty acids (PUFAs) have multiple double bonds, making them more reactive. PUFAs are divided into omega-3 and omega-6 families, essential for human health due to their roles in inflammation regulation, cell membrane structure, and development. Monounsaturated fatty acids (MUFAs) can be taken from olive oil and nuts and are known to lower LDL cholesterol levels while maintaining HDL cholesterol, thus supporting cardiovascular health and other diseases [19,20,21,22]. MUFAs also have anti-inflammatory properties and enhance insulin sensitivity, contributing to improved blood glucose control. Polyunsaturated fatty acids (PUFAs), comprising omega-3 (e.g., EPA and DHA) and omega-6 (e.g., linoleic acid) fatty acids, play distinct roles in cellular function and inflammation regulation. Omega-3 PUFAs are critical for brain function, reducing inflammation, and supporting cardiovascular health, whereas omega-6 PUFAs are essential for skin health and reproductive system development, though they require balance with omega-3s to prevent excessive inflammation. Both MUFAs acid and PUFAs acid have been shown to possess antimicrobial properties by disrupting microbial cell membranes in addition to their well-established roles in cardiovascular health and anti-inflammatory processes [20,21,23].

In addition to lipids, the terpenes and flavonoids identified in the extract, such as carnosic acid, rosmanol, and kaempferol, further contribute to its antimicrobial potential. Terpenes, especially carnosic acid, have strong antimicrobial effects against a spectrum of bacteria and fungi, making them eligible for combating infections [24,25]. Flavonoids are mostly renowned for their antioxidant properties but also demonstrate antimicrobial activity by inhibiting bacterial growth and biofilm formation [26].

Further analysis using GC-FID confirmed that palmitic acid was the most abundant fatty acid in the extract [18]. Based on the findings of antimicrobial activity from palmitic acid [23], and previously demonstrated antimicrobial activity from *C. linum* extract [27], an in vitro and in silico framework was set up to inspect the antimicrobial potential of the extract.

This study aimed to exploit the circular bioeconomy to propose plant-based by-products, in this case from *C. linum*, as valid sources of bioactive compounds showing antibacterial properties, through a combined in vitro and in silico pipeline.

## 2. Results

### 2.1. Evaluation of the Antibacterial Activity of the C. linum Extract

MIC values were determined and are shown in Table 1. The extract showed antibacterial activity on *E. faecalis* ATCC 29212 (MIC, 32 μg/mL), proving the antimicrobial activity of the alga extract against this strain. However, the extract was not active on the other tested strains. Indeed, an MIC value of 256 μg/mL was measured with *S*. *aureus* ATCC 25392, and even higher values were obtained with *E. coli* strain ATCC 25922 and *P. aeruginosa* ATCC 27853. Vancomycin and colistin (concentrations range: 32–0.015 μg/mL) were used as antibiotic controls for *E. faecalis* and *S. aureus*, and for *E. coli* and *P. aeruginosa*, respectively.

Although the extract showed a rather narrow spectrum of antibacterial activity with a MIC value of 32 μg/mL, the observed activity on the clinically relevant opportunistic pathogen *E. faecalis* was deemed interesting compared to what was observed for other seaweed extracts [28].

### 2.2. Multiple Sequence Alignments

The *in silico* analysis revealed that the *E. faecalis* strain was susceptible to the *C. linum* extract, which mostly contains fatty acids. In contrast, no vulnerability was observed in *P. aeruginosa*, *S. aureus*, or *E. coli* following treatment with the extract.

Considering the exclusive activity of the extract against *E. faecalis*, the potential *E. faecalis* targets interacting with our compound were identified. Therefore, a multiple sequence alignment (MSA) was performed between the full set of proteins from *E. faecalis* and the “Non-redundant protein sequences (nr)” database of *P. aeruginosa*, *S. aureus*, and *E. coli* to individuate the *E. faecalis* sequences yielding no homology with those of the other strains.

An in-house Python script analyzed 150,305,287 sequence alignments and pinpointed 38 *E. faecalis* protein sequences that had adequate coverage and showed less than 20% similarity/identity, indicating the non-homology. These sequences were further examined in the UniProt database, and any sequences labeled as “obsolete” were excluded. Ultimately, the endocarditis and biofilm-associated pilus tip protein EbpA (EbpA) was identified as the sole non-homologous *E. faecalis* sequence.

### 2.3. Molecular Modeling and Docking Simulation

**Figure 1:**
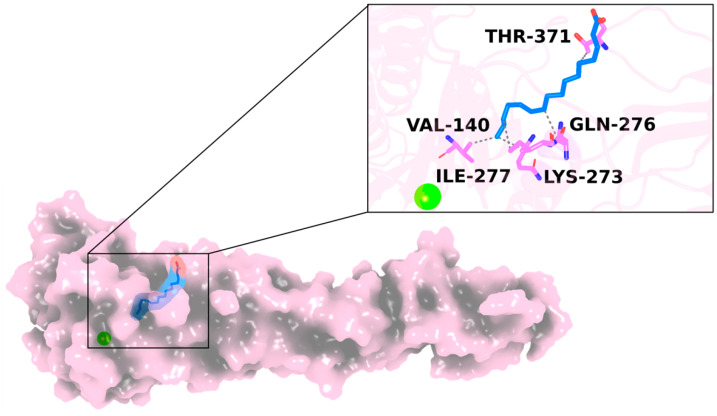
Representation of the docked pose of palmitic acid/EbpA complex. The EbpA 3D structure is depicted in a pink cartoon/surface, while the magnesium ion is reported as a green sphere. The interaction network of palmitic acid (in blue) is in complex with EbpA after the docking simulation. The binding residues forming hydrophobic interactions (gray dashed lines) and hydrogen bonds are shown as sticks, respectively.

The target 3D structure was generated and optimized using AlphaFold and molecular modeling tools. AlphaFold provided five distinct 3D structures by applying different weights and ranked them based on their mean pLDDT score. The first model, which exhibited the highest confidence in the predicted 3D structures, was selected as the reference target, and the pilus adhesin, the SpaC 3D structure of *Lactobacillus rhamnosus*, was used as a template for the molecular modeling to optimize its structure, solving all structural gaps and steric hindrances. Palmitic acid was evaluated against the target using in silico docking simulation. The docking results indicated that the complex EbpA/palmitic acid was favorable, with a binding free energy of −5.2 kcal/mol. Interaction network analysis highlighted that palmitic acid established hydrophobic interactions within the target’s binding pocket (Figure 1). Moreover, it formed hydrogen bonds with the residue Arg-113, whilst all the other residues were involved in a strong hydrophobic interaction network. These findings suggest that the compounds could spontaneously bind to *E. faecalis* EbpA.

### 2.4. cMD: EbpA/Palmitic Acid Complex (RMSD and Total Interaction Energy)

The interaction between the target and compound was analyzed by examining structural and energy characteristics through continuous molecular dynamics (cMD) simulations for both the complex and the free EbpA state.

**Figure 2:**
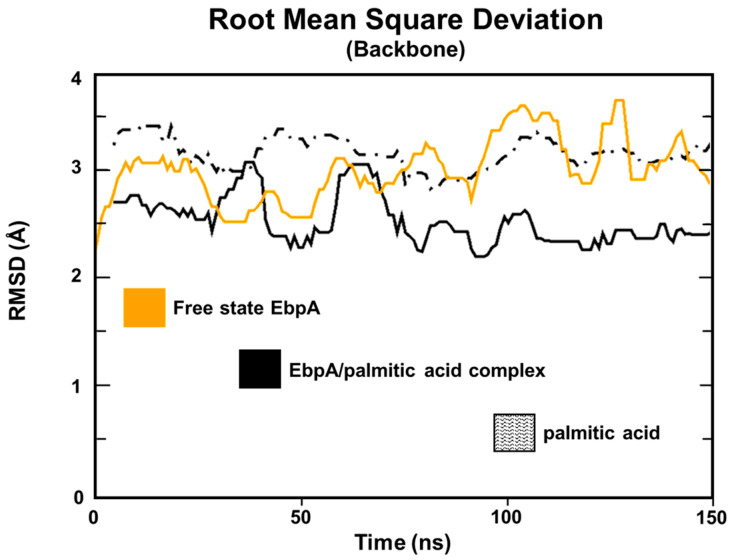
RMSD trends. RMSD profiles were evaluated for the free state and EbpA/ellagic acid backbone along the entire MD run.

The structural integrity of the target backbone was evaluated by calculating RMSD values for both the EbpA/palmitic acid complex and free EbpA. RMSD analysis revealed good structural stability. The EbpA/palmitic acid complex showed a stable RMSD trend of 2.5 Å and the free EbpA state reported a trend of 3 Å. The binding poses of palmitic acid within the target binding pocket remained stable, with an RMSD value of 3 Å along the entire MD run, confirming the reliability of the initial docking pose (Figure 2). To further support the docking and cMD simulation results, we evaluated the target/compound interaction energy. The energy analysis indicated that the compound spontaneously bound to the target with a total interaction energy of −60.0 ± 2.7 kcal/mol (Figure S2).

The involved binding residues in the interaction with the palmitic acid were further dissected through an evolutionary approach to define the conservation of residues along the protein evolution. Notably, the MSA analysis revealed that all the involved residues were fully conserved within the *Enterococcus* family, thereby confirming their relevance in the protein structure/function.

### 2.5. cMD: EbpA/Ellagic Acid Complex Interaction Network and Occupancy Analysis

**Figure 3:**
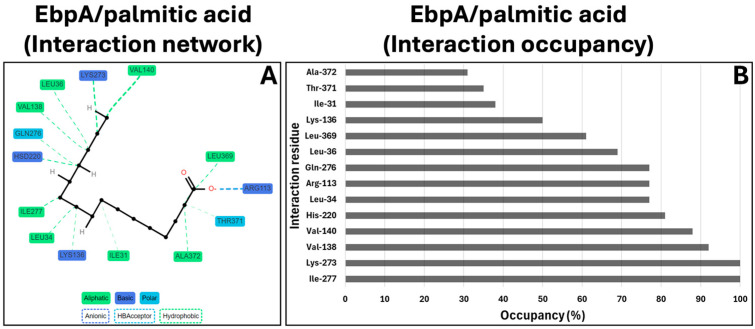
Interaction network and MD interaction network occupancy. (**A**) The 2D interaction network among target binding residues and palmitic acid (in black). The involved target residues are labeled as aliphatic (in green), basic (in dark blue), and polar (in light blue). The hydrogen bonds and hydrophobic interactions are represented as light blue and green dashed lines, respectively. (**B**) EbpA/palmitic acid MD interaction network occupancy. The occupancy percentage and the interaction residues are reported on the *x*- and *y*-axes, respectively. The occupancy displays the presence of each residue throughout the entire MD run.

To investigate the potential inhibitory effects of palmitic acid in its complex with the target, the dynamic interaction network during the molecular dynamics (MD) simulation was deeply dissected. The Prolif tool identified various types of interactions between the target and compound and assessed the occupancy of each interaction. The interaction network analysis revealed a range of polar and non-polar interactions (Figure 3A) with notably high occupancy values. Specifically, palmitic acid established hydrophobic interactions and hydrogen bonds. Figure 3 illustrates the interaction type and the binding residues, for which the occupancy percentage is also reported.

## 3. Discussion

Plants, including algae, hold significant potential in the pharmaceutical and medicinal fields, given their ability to produce a wide range of secondary bioactive metabolites. This arises from their adaptation to ever-changing environmental conditions, and it becomes even more important for algae, which possess the ability to survive fluctuating environments. Consequently, within the same species, metabolite production can vary significantly [3], rendering them ideal candidates for screening bioactive compounds with numerous biological activities.

Antimicrobial resistance is a universal issue advocating the design of new antimicrobial agents. Indeed, the worrying levels of resistant bacteria affect the efficacy of available antibacterial therapies and represent an increasingly important public health issue [29]. *E. faecalis* is a Gram-positive bacterium responsible for hospital-acquired infections (HAIs) such as urinary tract infections (UTIs), bacteremia, valve endocarditis, and wound infections [30,31]. Treatments for endocarditis, bacteremia, and UTIs typically involve combination therapy with cell wall-active agents like β-lactams and aminoglycosides [30].

However, *E. faecalis* showed a decreased susceptibility to penicillin and ampicillin [32] and intrinsic low-level aminoglycosides resistance, leading to worrying and growing antimicrobial resistance levels, together with an increasingly important concern in the hospital setting [32,33].

Several approaches to studying the antimicrobial activity of bio-compounds extracted from natural wastes were successfully applied, driving interest in the industry of drugs, cosmetics, nutritional supplements, and molecular probes. 

*C. linum*, an invasive alga from the Orbetello Lagoon in Tuscany, Italy, represents a significant ecological challenge and an opportunity for innovative resource utilization. Often regarded as special waste, its management incurs substantial costs. However, emerging evidence highlights its remarkable anti-inflammatory properties [18] and intriguing antimicrobial potential, which could be transformative for aquaculture practices [34,35].

This study took a groundbreaking approach by integrating both in vitro and in silico methodologies to comprehensively evaluate the antimicrobial properties of a hydroalcoholic extract of *C. linum*.

Our previous work unraveled its chemical composition and detected chemical classes such as lipids, amino acids, terpenoids, and flavonoids [18]. The detected lipids were as follows: palmitin (retention time: 41.79 min), oleic acid (retention time: 50.271 min), stearidonic acid (retention time: 24.921 min), hydroxymyristic acid (retention time: 40.396 min), hydroxylauric acid (retention time: 35.009), dihydroxypalmitic acid (retention time: 37.957 min), 9(10)-epoxyoctadecadienoic acid (retention time: 38.428 min), hydroxylinoleic acid (retention time: 40.866 min), lauramide (retention time: 35.481 min), and 8-pentadecenal (retention time: 41.627 min). Among the amino acids, the matched metabolites were valine (retention time: 2.043 min), norleucine (retention time: 1.877 min), and thymine (retention time: 8.042 min). The detected terpenoids were carnosic acid (retention time: 31.801 min), rosmanol (retention time: 28.817 min), and methyl dehydroabietate (retention time: 40.729 min). The last chemical class, terpenoids, included kaempferol (retention time: 12.302 min), apigenin (retention time: 32.899 min), and 3′-O-Methylequol (retention time: 23.484 min) [18].

To evaluate the effectiveness of ethanol/water extraction on the bioactive components of the crude extracts, extensive analyses were carried out by Nuclear Magnetic Resonance (NMR) spectroscopy and Ultra-Performance Liquid Chromatography coupled with Tandem Mass Spectrometry (UPLC-MS/MS). The NMR analysis revealed a notable presence of saturated and unsaturated fatty acids, characterized by wide peaks with higher intensities than polar metabolites. While peaks indicative of amino acids, sugars, and aromatic compounds were also detected, their concentrations were considerably low [18].

Building on these early results, the subsequent profiling of the *Chaetomorpha linum* extract (CLE) via UPLC-MS/MS led to identifying 19 metabolites, mostly lipids (free fatty acids and their derivatives). Among these were monounsaturated fatty acids (MUFAs), such as oleic acid (18:1ω-9), and polyunsaturated fatty acids (PUFAs), such as stearidonic acid (18:4ω-3).

Fatty acids are crucial for numerous biological processes, maintaining membrane fluidity, providing energy storage, and acting as precursors for bioactive lipids involved in inflammation and immunity. MUFAs are renowned for their role in cardiovascular health, providing anti-inflammatory effects and improving cholesterol levels. In contrast, PUFAs are involved in anti-inflammatory processes and chronic disease risk reduction, such as heart disease and diabetes [20,21].

Specifically, the matched compounds identified from the analysis included palmitic acid, its monoacylglycerol (MAG)-derivative (palmitin), hydroxy-derivatives of saturated fatty acids (hydroxymyristic acid, hydroxylauric acid, and dihydroxypalmitic acid), PUFA 18:3n-3 Alpha-linoleic acid-derived oxylipins (9(10)-EpODE, hydroxylinoleic acid). Oxylipins are signaling molecules derived from the oxidation of PUFAs, and they play a central role in regulating inflammation and immunity, acting as mediators in the resolution of inflammation [35].

Other matched compounds were a fatty acid amide (lauramide), and a fatty aldehyde (8-pentadecenal), associated with cell signaling and defense mechanisms against environmental stressors, respectively [36].

Amino acids (valine, norleucine, and thymine) were also identified. They are the well-known building blocks of proteins, crucial in metabolic pathways, muscle repair, and immune response regulation. For example, branched amino acid supplementation before squat exercises has been shown to delay onset muscle soreness [37].

To conclude, terpenes (carnosic acid, rosmanol, and methyl dehydroabietate), and flavonoids (kaempferol, apigenin, and 3′-O-Methylequol) were also detected through UPLC-MS/MS.

Terpenes possess antioxidant, anti-inflammatory, and antimicrobial properties against resistant pathogens [24]. Additionally, carnosic acid was largely studied for its neuroprotective and anti-cancer activities. [25]

Instead, flavonoids are primarily known for their antioxidant properties and protect cells from oxidative stress [38].

The additional characterization of the *C. linum* extract was achieved in our previous work through Gas Chromatography with Flame Ionization Detection (GC-FID) [18]. This reinforced the precedent findings and established palmitic acid (16:0) as the predominant fatty acid (52.18%), followed by myristic acid (14:0) (20.46%) and oleic acid (18:1ω-9) (11.70%). However, the *C. linum* phytochemical composition of these fatty acids varies depending on the location, extraction methods, ecological conditions, life cycles of the algae, and seasonal changes.

Given the predominance of fatty acids in the extract and their documented antimicrobial activity, the extract’s potential as an antimicrobial agent was further explored through in vitro and in silico studies, setting the basis for its application as an innovative natural antimicrobial.

This novel investigation deepened the understanding of the algae’s bioactive compounds while also exemplifying the principles of circular bioeconomy. By harnessing biological waste to create sustainable products, this research paves the way for innovative solutions to mitigate environmental impact while fostering economic viability in aquaculture. This exploration opens new avenues for the practical application of invasive species, turning a potential threat into a valuable asset.

We therefore tested the antibacterial activity of our *C. linum* extract on various bacterial strains, investigated the extract further to identify the extract’s compound responsible for bacterial growth inhibition, and subsequently searched for potential targets allowing us to rationalize the observed results.

First, the determination of the in vitro minimum inhibitory concentration (MIC) on a panel of both Gram-positive (*E. faecalis* and *S. aureus*) and Gram-negative (*E. coli* and *P. aeruginosa*) bacteria revealed a rather potent antibacterial activity on *E. faecalis* (MIC, 32 μg/mL), but not on the other tested strains (MIC values, ≥256 µg/mL). Consequently, the extract’s antimicrobial effect likely results from interactions between the bioactive compound palmitic acid and a specific target found exclusively in *E. faecalis* but that is absent in the other strains.

Through the multiple sequence alignment of the *E. faecalis* proteome, the “endocarditis and biofilm-associated pilus tip protein EbpA” was identified.

The 3D structure of EbpA was generated using AlphaFold. Computational screening subsequently indicated palmitic acid as a potential antimicrobial agent, with it being the most abundant in the extract. The docking simulation and total interaction energy unraveled the ligand/target interactions, with a good binding energy value. Additionally, the docking simulation showed that palmitic acid was bound between the two domains, forming the EbpA binding region. This region is involved in the binding to the intestinal mucin and collagen, necessary for successful host colonization by bacteria [39]. This would therefore explain the inhibitory effect of *C. linum* extract on bacterial growth. The MSA analysis confirmed the ability of the palmitic acid to bind to highly conserved EbpA residues.

## 4. Materials and Methods

### 4.1. Preparation of Chaetomorpha linum Extract (CLE)

In May 2021, *Chaetomorpha linum* was harvested and the extract was obtained. The algae *C. linum* (Müller) Kützing was gathered from the Orbetello Lagoon in Tuscany, Italy, and then washed with water to eliminate salt and debris. Once thoroughly cleaned, the material was dried in an oven at 55 °C until it reached a stable weight. It was then ground into a fine powder, which was later extracted using a mixture of ethanol and water (70:30 *v*/*v*) with a sample-to-solvent ratio of 1:10 (g/mL) and heated to 80 °C for three hours. After separating the supernatant from the remaining biomass and filtrating, rotary evaporation removed the solvent. The remaining aqueous solution was freeze-dried, yielding a dry extract. This extraction process was repeated twice. After extraction, 100 mg of the dry extract was dissolved in 1 mL of 100% DMSO, creating a 100 mg/mL CLE stock solution, which was then aliquoted and stored at −32 °C for further analysis. All of the procedures above mentioned were carried out in our previous work and all other details are reported in the study by Frusciante L. et al. [18].

The CLE was analyzed by UPLC-MS/MS, identifying 19 metabolites using Compound Discoverer 3.3 software, which utilized ChemSpider and mzCloud databases for data processing. Most of the metabolites detected in CLE were lipid-based, including free fatty acids (e.g., MUFA 18:1ω-9 oleic acid and PUFA 18:4ω-3 stearidonic acid) and the related fatty acid derivatives. Notably, a palmitic acid monoacylglycerol (MAG) derivative (palmitin) was identified, along with three hydroxyderivatives of saturated fatty acids (hydroxymyristic acid, hydroxylauric acid, and dihydroxypalmitic acid). Additionally, two PUFA 18:3n-3 alpha-linoleic acid-derived oxylipins (9(10)-EpODE and hydroxylinoleic acid), a fatty acid amide (lauramide), and a fatty aldehyde (8-pentadecenal) were detected. Other identified metabolites included amino acids (valine, norleucine, and thymine), terpenes (carnosic acid, rosmanol, and methyl dehydroabietate), and flavonoids (kaempferol, apigenin, and 3′-O-Methylequol) [18].

### 4.2. In Vitro Antibacterial Susceptibility Testing

The bacterial strains were obtained and cultured as previously reported [15].

*Enterococcus faecalis* ATCC 29212, *Staphylococcus aureus* ATCC 25392, *Escherichia coli* ATCC 25922, and *Pseudomonas aeruginosa* ATCC 27853 were obtained from the American Type Culture Collection (Manassas, VA, USA) and kept at −80 °C in Brain–Heart Infusion (BHI) medium containing 30% glycerol. The bacteria were routinely grown aerobically on Mueller–Hinton agar plates at 37 °C for 18–24 h.

Minimum Inhibitory Concentrations (MICs) of the extracts were determined in triplicate using the microdilution broth method in Mueller–Hinton medium, with a bacterial inoculum of 5 × 10⁴ CFU/well, following the Clinical Laboratory Standards Institute (CLSI) guidelines. The extracts were dissolved in DMSO to a final concentration of 50 mg/mL and then diluted in the culture medium (final concentration of 1024 µg/mL). In brief, serial two-fold dilutions of the extract were prepared in a 96-well plate (final volume per well: 90 µL), followed by the addition of 10 µL of a bacterial suspension, prepared extemporaneously, containing 5 × 10⁶ CFU/mL. The tested extract concentrations ranged from 512 to 0.25 µg/mL. The final concentration of DMSO in the assay did not exceed 1%. Growth controls with up to 1% DMSO were performed in parallel, with no observed inhibition in any of the tested organisms. MICs were recorded after 18 h of incubation at 35 ± 2 °C. Vancomycin and colistin, at concentrations ranging from 32 to 0.015 µg/mL, served as antibiotic controls for *E. faecalis* and *S. aureus*, or *E. coli* and *P. aeruginosa*, respectively.

### 4.3. In Silico Methods

#### 4.3.1. Sequence Resources and Multiple Sequence Alignment

The primary protein structures of the *E. faecalis* ATCC 29212 strain (197 sequences) were downloaded in FASTA format from the NCBI genome database (NCBI RefSeq assembly: GCF_000742975.1). In contrast, the protein sequences for *P. aeruginosa* (110,903 sequences), *S. aureus* (108,451 sequences), and *E. coli* (543,617 sequences) were retrieved from the “Non-redundant protein sequences (nr)” database, which houses nearly all available protein sequences at NCBI [40]. The Blastp tool enabled a multiple sequence alignment (MSA) between the *E. faecalis* sequences and the combined sequences of *P. aeruginosa*, *S. aureus*, and *E. coli* (compiled into a single FASTA file with 762,971 sequences). This was carried out using the BLOSUM 62 matrix, with a word size of 6, an E-value of 0.01, and the flags -sorthits 3 and -sorthsps 0 to sort the MSA output based on identity and E-value, respectively; all other settings remained at their default values [40].

The MSA process produced 150,305,287 sequence alignments, from which an in-house Python script was used to extract *E. faecalis* sequences (38 in total) that showed no identity or similarity with the other bacterial strains, with identity/similarity values lower than 20%. These 38 *E. faecalis* sequences were further searched in the UniProt database [41], and any sequences marked as “obsolete” were discarded, leading to the retainment of only one sequence: “endocarditis and biofilm-associated pilus tip protein EbpA” (NCBI Reference Sequence: WP_148304834.1).

The BLASTp tool performed MSA between the EbpA primary structure and the entire “non-redundant protein sequences (nr)” database among *Enterococcus* (TAXID: 1350), excluding *E. faecalis* (TAXID: 135). The Skylign tool then analyzed the MSA results to define the conservation of residues along the protein evolution.

#### 4.3.2. Molecular Modeling and Structure Optimization

The 3D structure of the *E. faecalis* endocarditis and biofilm-associated pilus tip protein EbpA was generated using AlphaFold and reported in Figure S1 [42], with the NCBI Reference Sequence WP_1483048.1 as the target. AlphaFold produced five distinct 3D structures by applying different weights and ranked them based on their mean pLDDT score. This uncertainty metric ranges from 0 to 100, where 0 indicates low uncertainty, and 100 indicates high uncertainty (with lower values being better). The first model, which had an uncertainty value of 2.81% and a mean pLDDT of 85.75, was selected as the best representation, indicating high confidence in the predicted 3D structure. A Mg^2+^ ion was incorporated into the model through structural superimposition, using the “Crystal structure of pilus adhesin, SpaC from *Lactobacillus rhamnosus* GG—open conformation” from *Lacticaseibacillus rhamnosus* (PDB code: 6M3Y) as a template [39].

#### 4.3.3. In Silico Docking and Molecular Dynamics Simulations

The 3D structure was optimized using PyMOD3.0 [43] with MODELLER 10.5 and validated through PROCHECK [44,45] to prevent errors during the molecular dynamic (MD) simulations. The parameters of the force field were assigned by CHARMM-GUI platforms [45]. GROMACS 2019.3 [46] carried out the MD run as suggested in previous works [47,48], where the structure was enclosed in a triclinic box filled with TIP3P water molecules and counter ions to neutralize the net charge of the system. The system was minimized by 5.000 steps using the steepest descent algorithm to a minimum energy with forces less than 100 kJ/mol/nm [49].

To detect a potential palmitic acid-binding region within EbpA, first, a blind docking was performed. The results pointed out that palmitic acid was mainly located within a hydrophobic region of the protein between the von Willebrand factor type A (vWA) and Collagen Adhesin A (CnaA) domains. Thus, a rational docking was performed by creating a box which included the binding region previously identified.

Docking simulation occurred between palmitic acid, the most abundant compound in the CLE, and EbpA. The 3D structure of palmitic acid was obtained from the PubChem database [50,51] (with compound CID: 985) and downloaded in sdf format. A box with dimensions of 25 × 25 × 25 Å was created around the sensing region of the target using DockingPie2.0, implemented in PyMOL3.0 using Autodock/Vina as a docking tool [52,53]. The docking simulation was set with an exhaustiveness of 32, selecting only the binding poses with a RMSD lower than 2 Å. All other parameters were set as default. The P.L.I.P. Tool [54,55] provided the interaction network. ClustalW [56] performed the sequence alignment.

Molecular dynamics (MD) analyses were carried out as previously described [47,48] using GROMACS 2019.3, with graphs generated by GRACE and visualizations created with PyMOL 3.0.

## 5. Conclusions

Our research was conducted within the circular bioeconomy concept. Natural sources, in this specific case plants, can be reused for their ability to produce bioactive compounds against molecules implicated in disease development, such as bacterial infections. Through a joined in vitro and in silico approach, novel bioactive compounds and their specific targets were individuated, enhancing the efficiency and cost-effectiveness of the process.

In vitro data indicated that the extract from *C. linum* exhibited antimicrobial activity primarily on *E. faecalis*. The in vitro antibacterial susceptibility assays demonstrated the antibacterial activity of the extract, with a MIC value of 32 μg/mL on this clinically relevant opportunistic pathogen. Afterwards, the specific bacterial target was identified, and the interaction between the extract’s major compound (palmitic acid) and the target protein was explored using docking and molecular dynamics simulations. These findings, particularly the interaction between palmitic acid in the extract and the target protein EbpA of *E. faecalis*, can be utilized for future experimental validation through in vitro binding assays.

Palmitic acid was considered because it was the most abundant within the extract. The in silico results supported its potential bioactivity against its predicted *E. faecalis* target, which would be interesting to further investigate. However, this research aligns with circular bioeconomy principles, and the focus was not on proposing palmitic acid as a potential antimicrobial compound, but rather on utilizing the whole extract as an antimicrobial source against *E. faecalis*. Investigating the potential of the whole extract within a sustainable and integrated bioprocessing strategy would offer a sustainable and eco-friendly approach to investigating the biological activity of compounds derived from plant by-products. On the plus side, time, costs, and chemical waste dwindled, and the environmental impact of pollutants was lessened. Following adequate further research, this seaweed extract could be employed in the research for new antimicrobial agents in different fields.

## Figures and Tables

**Table 1:** Evaluation of the minimum inhibitory concentration (MIC) of the *Chaetomorpha linum* extract (CLE) against the reference strains of *E. faecalis*, *S. aureus*, *E. coli*, and *P. aeruginosa* (see Materials and Methods for details). The MIC values of the control antibiotics (vancomycin or colistin, showing their expected activity on Gram-positive and Gram-negative bacteria, respectively) are also shown. The MIC values represent the mode obtained from three independent experiments.

Organism	MIC (μg/mL)		
	CLE	Vancomycin	Colistin
*E. faecalis*	32	2	- *^a^*
*S. aureus*	256	1	-
*E. coli*	512	-	0.06
*P. aeruginosa*	>512	-	0.12

*^a^*, not determined.

## Data Availability

The original contributions presented in the study are included in the article/Supplementary Materials; further inquiries can be directed to the corresponding author.

## Supplementary Materials

The following supporting information can be downloaded at https://www.mdpi.com/article/10.3390/md22110511/s1. Figure S1. Overview of the 3D structure of EbpA; Figure S2. Total interaction energy of EbpA in complex with PA.
