# Supplementary figures and images for: Chaetomorpha linum Extract as a Source of Antimicrobial Compounds: A Circular Bioeconomy Approach
Source: Mar Drugs. 2024 Nov 13;22(11):511. doi: 10.3390/md22110511 (PMC11595338; doi:10.3390/md22110511)

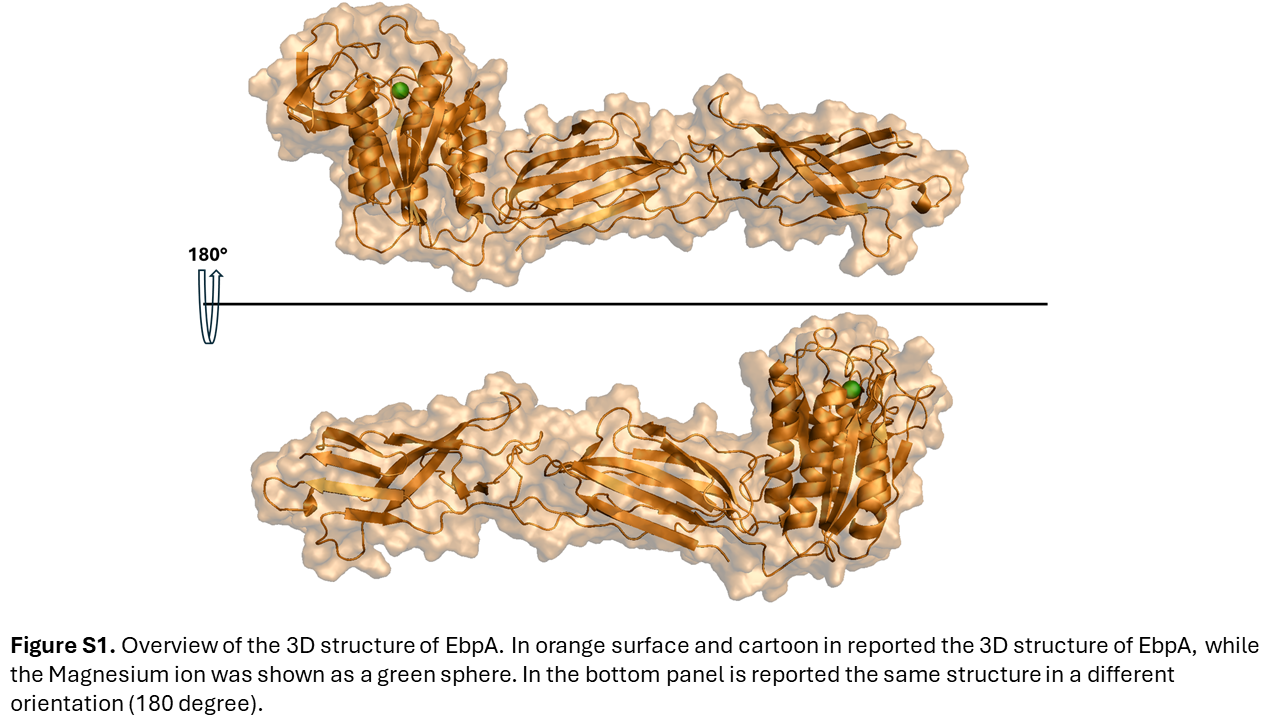

Supplement: Supplementary file 1 [file marinedrugs-22-00511-s001.zip › Figure S1.png]

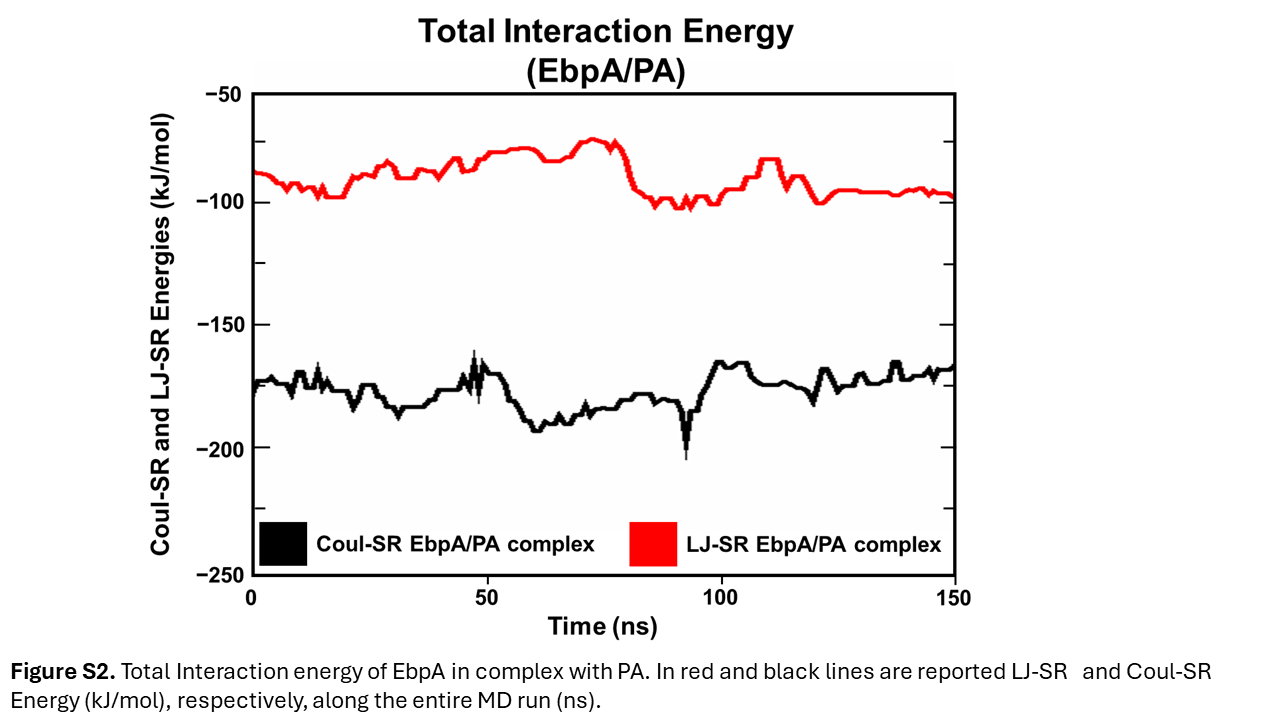

Supplement: Supplementary file 1 [file marinedrugs-22-00511-s001.zip › Figure S2.png]
